# Does Serum Anti-tissue Transglutaminase Level Predict Duodenal Histopathology and Clinical Manifestations of Celiac Disease in Adults?

**DOI:** 10.34172/mejdd.2025.429

**Published:** 2025-07-30

**Authors:** Reza Gholamrezaei, Atefeh Vaezi, Fatemeh Maghool, Nahid Jamali, Mohammad Hassan Emami

**Affiliations:** ^1^Isfahan University of Medical Sciences, Isfahan, Iran; ^2^Cancer Prevention Research Center, Isfahan University of Medical Sciences, Isfahan, Iran; ^3^Poursina Hakim Digestive Diseases Research Center, Isfahan University of Medical Sciences, Isfahan, Iran; ^4^School of Health Management and Information Science, Iran University of Medical Sciences, Tehran, Iran; ^5^Iranian Celiac Association (ICA), Isfahan, Iran

**Keywords:** Celiac disease, Clinical manifestations, Tissue anti-transglutaminase, Marsh system classification

## Abstract

**Background:**

Celiac disease (CD) is an autoimmune disorder triggered by gluten ingestion. This study aimed to evaluate the correlation between anti-tTG antibodies and histopathology, as classified by the Marsh system, and clinical manifestations.

**Methods:**

This retrospective cross-sectional study analyzed the records of 346 patients with confirmed CD from 2010 to 2021. Anti-tTG levels, clinical manifestations, body mass index (BMI), and pathological results were reviewed.

**Results:**

This study included 346 patients (mean age 26.5±16.6 years; 56.4% female). Most patients (53.2%) had atypical CD, and normal BMI was prevalent (57.1%). Common symptoms included anxiety (70.2%), fatigue/weakness (64.2%), borborygmi (53.2%), abdominal pain (51.2%), and bloating (46.8%). Disease severity analysis revealed 75.1% had Marsh III, while 15.9% had normal/Marsh I and 9.0% had Marsh II. Anti-tTG levels were significantly higher in advanced Marsh classes (Marsh III: 169.27±180.17 vs. normal/Marsh I: 70.94±116.45, *P*<0.001) and in typical CD (180.44±216.22) compared to atypical CD (126.20±121.72, *P*=0.005). Diarrhea, arthralgia, and osteoporosis/osteomalacia showed significant correlations with anti-tTG levels (*P*<0.05). ROC analysis for anti-tTG in diagnosing Marsh II or higher yielded an AUC of 0.733.

**Conclusion:**

Anti-tTG demonstrated moderate diagnostic accuracy for advanced duodenal damage, highlighting its utility as a biomarker in CD. Larger studies are needed to validate these findings further.

## Introduction

 Celiac disease (CD) is an autoimmune enteropathy that occurs in genetically susceptible individuals after they consume gluten, which is found in barley, wheat, and rye.^1^ The global prevalence of CD is approximately 0.7% to 1.4%, based on serological and pathological findings. In Iran, it is approximately 0.79% to 0.83%.^2,3^

 CD may be asymptomatic or symptomatic, presenting with a range of gastrointestinal and extraintestinal symptoms.^4^ Typical clinical symptoms include diarrhea, steatorrhea, and weight loss (consistent with malabsorption), while atypical clinical symptoms include fatigue, dyspepsia, unexplained abdominal pain, constipation, anemia, osteoporosis, growth impairment, and other extraintestinal.^4,5^ The probability of having CD is significantly higher among patients with typical clinical manifestations than those with atypical clinical manifestations.^6^

 In most cases, the diagnosis of CD is based on serological and histological findings, as well as a response to a gluten-free diet (GFD). However, approximately 10% of patients may not be diagnosed due to the lack of correlation between serological and histological findings and clinical symptoms.^7,8^ For serological evaluation, the anti-endomysial antibody test has 100% specificity. However, due to its reliance on immunofluorescence and operator skill, it is more commonly used for diagnosing the disease in children without needing a biopsy. In contrast, the preferred test in adults is the anti-tissue transglutaminase antibody (anti-tTG) assay, which has high sensitivity and specificity.^9,10^

 The most common classification system for determining the severity and degree of mucosal damage is the Marsh classification, which categorizes the severity of mucosal damage into three grades based on mucosal lymphocytosis, crypt hyperplasia, and villous atrophy.^8^

 Although recent studies have supported omitting upper gastrointestinal (GI) endoscopy with duodenal biopsies to diagnose CD in adults, more studies and meta-analyses are needed, and the definitive diagnosis of CD is still based on endoscopic duodenal biopsy and histopathological examination.^11^ Despite the high cost to the healthcare system, the results of pathological tests largely depend on the observer’s experience. Therefore, understanding the quantitative relationship between serological and histopathological findings may provide a reliable method to determine the optimal serological level, thereby reducing the costs of endoscopy and biopsy, as well as the dependence on the observer. However, research in this area is limited and has not been conducted in Iran. Therefore, this study aimed to investigate the relationship between anti-tissue transglutaminase antibodies and histopathology, as classified by the Marsh system, and clinical manifestations.

## Materials and Methods

 This retrospective cross-sectional study was conducted at the Iranian Celiac Association (ICA) and Poursina Hakim Gastroenterology Research Center of Isfahan. Data were gathered and analyzed from the patient’s health records in ICA, which were reliably collected between 2010 and 2021. Ethical Committee approval of Isfahan University of Medical Sciences (ID: IR.MUI.MED.REC.1400.768) was obtained. The study included 346 patients aged 18 years or older who had CD. Patients with a documented diagnosis of irritable bowel syndrome (IBS) or diarrhea-predominant IBS (IBS-D) were not included in the study, as the primary focus was on CD-related manifestations. The patients were selected through a non-random convenience sampling method. We reviewed patients’ health records and their clinical symptoms, BMI, tissue anti-transglutaminase serological test (IgA anti-tTG) results, and pathological test results (Marsh I, Marsh II, and Marsh III). Dental enamel defects, if documented, were also noted, as a dentist typically diagnoses them through clinical examination and imaging techniques such as radiography.

 Patients undergoing treatment with chemotherapy or immunosuppressive drugs were excluded from the study. Patients with IgA deficiency were also tested for IgG anti-tTG; if unavailable, they were excluded from the study. BMI categories were defined as < 18.5 for underweight, 18.5-24.9 for normal, 25.0-29.9 for overweight, and ≥ 30.0 for obesity.^12^

 The documented records of patients definitively diagnosed with CD based on serological tests, duodenal biopsy, genetic tests, and response to a GFD were analyzed to investigate the relationship between serological tests (IgA & IgG anti-tTG) and clinical manifestations (typical and atypical) as well as histopathology based on the Marsh classification. In this study, a level of anti-tTG higher than 18 U/mL, as determined by enzyme-linked immunosorbent assay (ELISA) testing using a commercial kit from the manufacturer (AESKU, Inc.), was considered positive.

 In patients with documented bone pain or suspected bone-related complications (e.g., osteoporosis or osteomalacia), the diagnosis was based on a clinical evaluation that included a detailed history of bone pain, fractures, or other related symptoms. Bone pain was defined as localized or generalized discomfort in the bones, often associated with conditions such as osteoporosis, osteomalacia, or vitamin D deficiency. For patients with suspected osteoporosis, bone mineral density (BMD) was assessed using dual-energy X-ray absorptiometry (DXA) scans, where available. Osteoporosis was defined as a T-score of ≤ -2.5 at the lumbar spine or femoral neck, according to the World Health Organization (WHO) criteria. Osteomalacia was diagnosed based on clinical symptoms, biochemical markers (e.g., low serum calcium, phosphate, and vitamin D levels), and, if available, radiographic findings such as pseudofractures or Looser zones. However, due to the retrospective nature of this study, BMD data and detailed biochemical markers were not available for all patients.

 Data were analyzed using SPSS software for Windows, version 24 (SPSS Inc.). Mean and standard deviation (SD) were used to describe quantitative variables, while frequency and percentage were used to describe qualitative variables. Furthermore, t-test and one-way ANOVA were used to compare means between groups, and the chi-square test was used to compare qualitative variables between groups. The receiver operating characteristic (ROC) curve was administered to calculate and define the accuracy of the suggested test. The sensitivity, specificity, positive likelihood ratio (PLR), and negative likelihood ratio (NLR) of serum levels of anti-tTG at cut-off points of 3, 6, 9, and 12 times the upper limit of normal (ULN) (18 U/mL) were calculated. The significance level was considered at *P* value < 0.05.

## Results

 In total, 346 patients with a mean ( ± SD) age of 26.5 ( ± 16.6) years were included in this study, of which 195 (56.4%) were female. Most of the patients (53.2%) presented with atypical CD. The normal BMI category was the most prevalent in our study population (57.1%), and fewer than 5% of the patients were obese. The most observed symptoms in our study patients were anxiety (70.2%), fatigue and weakness (64.2%), borborygmi (53.2%), abdominal pain (51.2%), and bloating (46.8%). Additionally, diarrhea was observed in approximately 20% of the patients. Regarding the severity of the CD, 55 (15.9%) of our study population had normal pathology or Marsh I, 31 (9.0%) had Marsh II, and 260 patients (75.1%) had Marsh III.

 As shown in Table 1, the mean ( ± SD) level of anti-tTG in the whole population was 151.6 ( ± 174.39). With the advancement of the CD Marsh classes, the level of anti-tTG significantly increased. In Marsh III, the mean ( ± SD) level of anti-tTG was 169.27 (180.17), whereas in those with normal or Marsh I, it was 70.94 ( ± 116.45) (*P* < 0.001).

**Table 1:** Association between demographic and baseline characteristics and stage of celiac disease (Marsh category)

**Variable**		**Total**	**Marsh category**			***P***
			**Nl or I 55 (15.9)**	**II 31 (9.0)**	**III 260 (75.1)**	
Age, mean (SD)	Year	26.53 (16.67)	29.93 (14.02)	26.82 (13.99)	25.78 (17.43)	0.24
Sex, N (%)	Male	151 (43.6)	25 (45.5)	17 (54.8)	109 (41.9)	0.28
	Female	195 (56.4)	30 (54.5)	14 (45.2)	151 (58.1)	
BMI, N (%)	Thin	80 (23.2)	16 (29.1)	10 (32.3)	115 (44.2)	0.31
	Normal weight	197 (57.1)	28 (50.9)	12 (38.7)	97 (37.3)	
	Over weight	53 (15.4)	8 (14.5)	7 (22.6)	38 (14.6)	
	Obese	15 (4.3)	3 (5.5)	2 (6.5)	10 (3.8)	
Anti-tTG Ab level (mean ± SD)		151.60 ± 174.39	70.94 ± 116.45	146.56 ± 174.42	169.27 (180.17)	< 0.001
Presentations types, N (%)	Typical	162 (46.8)	29 (52.7)	17 (54.8)	116 (44.6)	0.35
	Atypical	184 (53.2)	26 (47.3)	14 (45.2)	144 (55.4)	
Sign and symptoms						
Abdominal pain, N (%)		177 (51.2)	31 (56.4)	17 (54.8)	130 (50.0)	0.64
Abdominal discomfort, N (%)		131 (37.9)	28 (50.9)	13 (41.9)	91 (35.0)	0.08
Abortion, N (%)		13 (6.6)	1 (1.8)	1 (3.3)	12 (4.6)	0.62
Alopecia areata, N (%)		143 (41.3)	29 (52.7)	13 (41.9)	102 (39.2)	0.18
Amenorrhea, N (%)		31 (15.8)	8 (14.5)	4 (12.9)	20 (7.7)	0.21
Anxiety, N (%)		243 (70.2)	42 (76.4)	23 (74.2)	179 (68.8)	0.48
Anorexia, N (%)		79 (22.8)	15 (27.3)	5 (16.1)	60 (23.1)	0.50
Aphthous lesion, N (%)		108 (31.2)	19 (34.5)	10 (32.3)	80 (30.8)	0.86
Arthralgia, N (%)		114 (32.9)	23 (41.8)	9 (29.0)	83 (31.9)	0.32
Ascites, N (%)		1 (0.3)	0 ()	0 ()	2 (0.8)	0.72
Bloating, N (%)		162 (46.8)	33 (60.0)	16 (51.6)	114 (43.8)	0.08
Bone pain, N (%)		90 (26.0)	16 (29.1)	10 (32.3)	65 (25.1)	0.61
Borborygmi, N (%)		184 (53.2)	36 (65.5)	21 (67.7)	128 (49.2)	0.022
Cheilosis, N (%)		1 (0.3)	0 ()	0 ()	2 (0.8)	0.72
Coagulopathy, N (%)		2 (0.6)	0 ()	1 (3.2)	2 (0.8)	0.27
Constipation, N (%)		96 (27.7)	18 (33.3)	8 (26.7)	71 (27.5)	0.67
Dental enamel defect, N (%)		171 (49.4)	27 (49.1)	12 (40.0)	133 (51.2)	0.51
Depression, N (%)		129 (37.3)	23 (41.8)	14 (46.7)	93 (35.9)	0.41
DM-Type I, N (%)		7 (2.0)	0 ()	1 (3.3)	7 (2.7)	0.45
Epilepsy, N (%)		15 (4.3)	2 (3.6)	0 ()	14 (5.4)	0.38
Flatulence, N (%)		15 (4.3)	3 (5.5)	3 (1.0)	10 (3.8)	0.30
Hypertransaminasemia, N (%)		6 (1.7)	2 (2.6)	0 ()	5 (1.9)	0.51
Hypo/hyperthyroidism, N (%)		10 (2.9)	1 (1.8)	3 (10.0)	7 (2.7)	0.08
Jaundice, N (%)		41 (11.8)	4 (7.3)	4 (12.9)	34 (13.1)	0.48
Malodor stool or gas, N (%)		125 (36.1)	23 (41.8)	14 (45.2)	89 (34.2)	0.32
Nausea, N (%)		89 (25.7)	17 (30.2)	6 (20.0)	67 (25.8)	0.53
Osteoporosis/malacia, N (%)		11 (3.2)	1 (1.8)	1 (3.3)	10 (3.8)	0.76
Skin lesion, N (%)		32 (9.2)	6 (10.9)	3 (10.0)	24 (9.2)	0.93
Short stature, N (%)		23 (6.6)	4 (7.3)	0 ()	20 (7.7)	0.29
Steatorrhea, N (%)		77 (22.3)	13 (23.6)	7 (22.6)	58 (22.3)	0.98
Vomiting, N (%)		15 (43)	0 ()	3 (10.0)	13 (5.0)	0.09
Weakness fatigue, N (%)		222 (64.2)	41 (74.5)	23 (74.2)	159 (61.2)	0.08
Weight loss, N (%)		81 (23.4)	7 (12.7)	6 (20.0)	69 (26.5)	0.08
Diarrhea, N (%)		75 (21.7)	13 (23.6)	9 (29.0)	54 (20.8)	0.54
Anemia, N (%)		119 (34.4)	21 (38.2)	9 (30.0)	90 (34.6)	0.75

BMI: Body mass index.

 The frequency of borborygmi was higher in higher socioeconomic classes (*P* = 0.022). Abdominal discomfort, abortion, and hypo/hyperthyroidism had *P* values very close to being significant (0.05), which may become significant in larger sample sizes. Some signs and symptoms, such as decreased libido, delayed puberty, delayed menarche, edema, hematuria, infertility, lymphadenopathy, and polyneuropathy, could not be statistically analyzed due to insufficient sample size. Baseline and demographic characteristics, clinical manifestations, and the sero-histological severity of CD are presented in Table 1.

 The mean ( ± SD) level of anti-tTG antibodies in patients with typical CD was 180.44 ( ± 216.22), which was higher than in patients with atypical CD, with 126.20 ( ± 121.72) (P = 0.005). Among the clinical manifestations, only diarrhea (*P* < 0.001), arthralgia (*P* = 0.03), osteoporosis/osteomalacia (*P* = 0.05), and weight loss (*P* = 0.06) showed a significant correlation with anti-tTG levels (*P* < 0.001). The remaining symptoms had no significant association with Anti-tTG levels (all *P* > 0.05).

**Figure 1:**
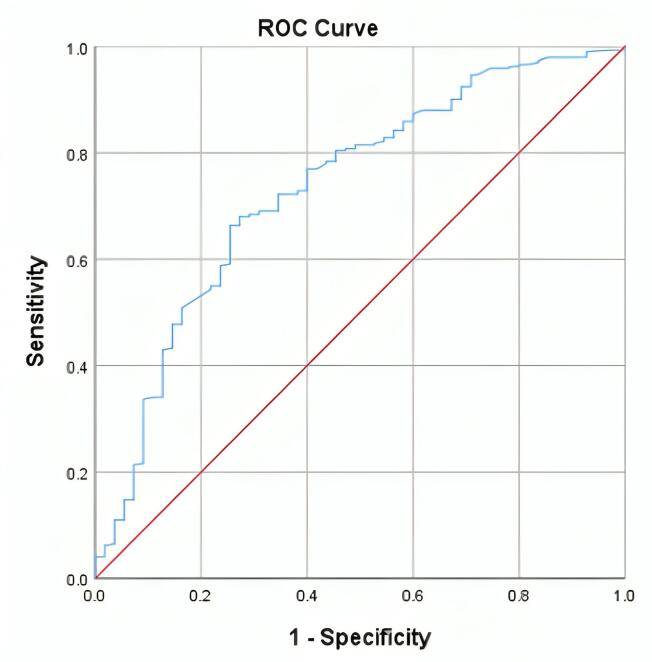
ROC Curve for diagnostic performance of Marsh grade 2 or greater. Area under the curve (AUC) = 0.733, Standard error = 0.038, *P* < 0.0001, 95% confidence interval (0.658 to 0.807), Null hypothesis: true area = 0.5

 According to Figure 1, the ROC curve analysis was used to assess the diagnostic accuracy of serum/cut-off levels of anti-tTG for diagnosing Marsh II duodenal damage or higher, and an area under the curve (AUC) of 0.733 was found. The sensitivity, specificity, PLR, and NLR of serum levels of Anti-tTG at threshold values of 3, 6, 9, and 12 times the ULN are presented in Table 2.

**Table 2:** Sensitivity, specificity, positive likelihood ratio, and negative likelihood ratio of anti-tTG normalized to the upper limit of normal (ULN) for the diagnostic performance of marsh grade 2 or higher

**Anti t-TG levels**			**Diagnostic performance**		
**Cut-off (U/mL)**	**ULN (multiples)**	**Sensitivity (%)**	**Specificity (%)**	**Positive LR**	**Negative LR**
> 54	3**×**	68.4	69.1	2.21	0.46
> 108	6**×**	50.2	83.6	3.06	0.60
> 162	9**×**	39.5	87.3	3.11	0.69
> 216	12**×**	26.8	90.9	2.95	0.81

## Discussion

 To accurately diagnose CD as a complex condition, a multidisciplinary approach is necessary. Although histology is considered a standard for diagnosing CD, these methods have many limitations. These limitations include the need for endoscopic biopsy, tissue preparation, and variability in the results based on the observer. Therefore, serological tests and clinical symptoms are required for accurate disease diagnosis.^13^ The European Society for Pediatric Gastroenterology, Hepatology, and Nutrition (ESPGHAN) guidelines recommend avoiding duodenal biopsy in children with anti-tTG levels greater than ten times the average, a positive EMA, and no clinical doubt. Guidelines for adults still recommend integrating serological and histological results.^14^

 Recent studies have further investigated the relationship between serological markers and histological findings in CD. Penny et al conducted a study to evaluate the predictive value of IgA tTG levels of ≥ 10 times the ULN in identifying adults with Marsh III lesions. Their results showed that IgA tTG titers of ≥ 10 times the ULN are strongly predictive of detecting intestinal changes indicative of CD. While sensitivity, specificity, positive predictive value, and negative predictive value varied across different cohorts, the overall findings support a no-biopsy approach for diagnosing adult CD in specific cases.^15^ This aligns with the ESPGHAN guidelines for children and suggests that a similar approach could also be applicable for adults with high anti-tTG levels.

 Rostami Nejad et al conducted a study to investigate the prevalence of CD antibodies among patients experiencing GI symptoms. They found that 3.7% of these patients tested positive for CD antibodies, which highlights the importance of recognizing atypical presentations of CD. The study also noted that non-specific GI symptoms, such as dyspepsia, are frequently observed in patients with atypical CD. Increased awareness of these presentations could enhance the identification of asymptomatic or atypically symptomatic patients.^16^ These findings underscore the importance of a comprehensive diagnostic approach that considers both serological and clinical factors, particularly for patients presenting with non-specific symptoms.

 Regarding the relationship between tissue damage and symptoms, our study found that borborygmi was directly related to the severity of epithelial damage in CD. The severity of tissue damage was significantly higher in patients with borborygmi. Similar or contrasting findings have been reported in previous studies.^17,18^ In the study by Ziv-Baran et al, which was conducted on children with a median age of 6 years, no significant relationship was found between the presence of symptoms, such as diarrhea and abdominal pain, and the severity of tissue damage based on the Marsh classification, as well as the level of antibodies. However, a significant relationship was observed between some indicators related to the severity of tissue damage and the presence of anemia. This study confirms that a high anti-tTG level is a crucial non-invasive measure that reflects both macroscopic and microscopic mucosal damage, potentially leading to anemia.^17^

 Note that adhering to a GFD can help control many symptoms, including those mentioned above, in patients with CD.^19^ Furthermore, in the long term, such a diet can significantly reduce the level of anti-tTG in patients with CD.^20^ Our study did not investigate patient adherence to GFD or other therapeutic measures. However, considering the points mentioned above, the relationship between the symptoms and the level of antibodies can be disrupted by a GFD, and this can significantly influence the results of laboratory studies and other similar studies. Therefore, GFD should not be initiated until a definitive diagnosis is made. Arthralgia can be observed in 26% of patients with CD and is considered an essential sign in these patients.^21^ Arthralgia in these patients may be due to vitamin D deficiency, juvenile idiopathic arthritis, or osteoporosis, or it may occur independently.^22^ In both children and adults, introducing a GFD supplemented with calcium and vitamin D can significantly improve these symptoms.^23^

 It appears that the severity of symptoms, geographical region, dietary habits, potential environmental pollutants, genetics, and demographic characteristics of patients under investigation can all influence the presentation of CD, which may manifest with various GI and non-GI symptoms, significantly impacting the patient’s quality of life. The most common symptoms observed in the studied patients were anxiety, fatigue, borborygmi, abdominal pain, and bloating. In addition, diarrhea was observed in approximately 20% of our patients. Various studies have shown that the prevalence of depression in patients with CD can range from 6% to 69%, and the prevalence of anxiety symptoms can range from 16% to 85%. These symptoms can be influenced by factors such as age, sex, environmental conditions, lifestyle, and disease duration.^24,25^ In Iranian studies, the prevalence of anxiety in patients with CD was close to 70%.^25^ The most common symptoms observed in these patients were abdominal pain, bloating, diarrhea, and fatigue.^6^ In another study, the most common symptoms in adults with CD were chronic fatigue, abdominal pain and bloating, anemia, headache, and diarrhea.^26^

 The association between the degree of duodenal tissue damage and the level of anti-tTG in children and adults has been found in previous studies and was also observed in the present study. Patients with severe tissue damage had significantly higher antibody levels.^27,28^ Furthermore, in adults, a diagnostic performance cut-off of more than 6.2 times the normal limit for anti-tTG showed improved sensitivity and specificity.^14^ However, our study showed no such cut-off level at which endoscopy and duodenal histology would be unnecessary. Larger studies or meta-analyses of the present studies may provide a more precise answer to this question.

 Although our study was conducted in a very experienced center, it had some limitations, including being performed in a single center with a limited sample size and a retrospective design. It is recommended that future studies be conducted in a multi-center setting to enhance the generalizability of the results. Additionally, the nature of histopathological tests and their dependence on the observer should be considered. Endoscopy, duodenal sampling, and histopathological studies should be performed in a center specializing in GI histopathology, as is the case in our center. The duration of signs, symptoms, and diet habits should also be considered in future studies.

 In general, experiencing chronic diarrhea and anemia or the following symptoms, such as anxiety, abdominal bloating, abdominal pain, feelings of weakness, and fatigue, should be considered highly suspicious symptoms for CD. It is also worth noting that the prevalence of typical and atypical symptoms varies across different populations.^26,29^

 Regional studies, such as the study in Isfahan, can be enlightening; however, due to the high cost and time required to achieve this goal, conducting meta-analyses of such studies is a quicker solution for summarizing various studies and reaching reliable results.

## Conclusion

 This study highlights the diverse clinical and serological presentations of CD, showing that anti-tTG levels are significantly associated with disease severity, being higher in typical cases than in atypical ones. Symptoms such as diarrhea, joint pain, and osteoporosis were correlated with anti-tTG levels.

 The diagnostic accuracy of anti-tTG for identifying advanced duodenal damage (Marsh II or higher) was moderate, with an AUC of 0.733. These results stress the importance of anti-tTG as a severity biomarker and suggest a need for larger studies to explore the links between clinical symptoms and serological markers in CD.
